# Youth in times of polycrises: the role of social support in connection with the depression and anxiety symptoms among young people in polish secondary schools during the COVID-19 threat and the outbreak of war in Ukraine

**DOI:** 10.3389/fpsyg.2025.1591218

**Published:** 2025-10-06

**Authors:** Karina Badura-Brzoza, Patryk Główczyński, Dominika Tatar, Paweł Dębski

**Affiliations:** Clinical Department of Psychiatry, Faculty of Medical Sciences in Zabrze, Medical University of Silesia, Katowice, Poland

**Keywords:** social support, adolescence, psychiatry, war, COVID-19

## Abstract

**Aim:**

To assess perceived social support and its relationship with symptoms of depression and anxiety among secondary school students in a crisis situation such was the outbreak of the Russian-Ukrainian war and the ongoing Covid 19 pandemic.

**Materials and methods:**

The study involved 1,456 students aged 14–19 (mean age 16.77 ± 1.33), including 776 girls and 680 boys. Data were collected during a single data collection period from February 2022 to May 2022. Participants completed the Multidimensional Scale of Perceived Social Support (MSPSS), Children’s Depression Inventory 2 (CDI-2), the State–Trait Anxiety Inventory (STAI-X1), and a sociodemographic questionnaire. Data were analyzed using non-parametric tests and correlation analyses.

**Results:**

Girls reported significantly higher levels of perceived social support from significant people and friends as well as higher symptoms of depression and anxiety, compared to boys. Negative correlations were found between perceived social support and symptoms of depression and anxiety in both genders.

**Conclusion:**

The conducted research indicates that perceived social support may play an important role in coping with symptoms of anxiety and depression in crisis situations.

## 1 Introduction

### 1.1 Social support: definition and significance

Social support encompasses all resources available to an individual, which are utilized in difficult situations to cope with stress arising from those circumstances (Cohen, 2004; Sikora, 2012; Lin et al., 1985). At every stage of life, people form more or less extensive social networks, maintained through bonds that connect them with others. Social support can have a direct impact on a person’s health at any point in their life regardless of the occurrence of challenging situations. It can also act as a moderator that positively influences well-being at the exact moment when a stressor occurs or immediately afterwards (Lin et al., 1985; Reicher, 1997; Yarchesky et al., 2001; Cohen and Hoberman, 1983). These two models are not mutually exclusive but rather complementary, offering a framework for understanding how specific aspects of social relationships influence mental health (Buszman and Przybyła-Basista, 2017). Research indicates that support networks are formed throughout the human lifespan and exist even when their activation is not immediately necessary. The most important networks are the natural ones, such as family, friends, close acquaintances, and social groups to which an individual belongs. Scholars emphasize the vital role of social support in shaping overall well-being and its significant effects on both physical and mental health (Uchino et al., 1996; Reblin and Uchino, 2008).

### 1.2 Adolescence and the role of social support

Adolescents constitute a group for which social support—particularly from peers—is of crucial importance. Maturation is inherently a challenging process, during which identity formation occurs alongside the development of social bonds. This period is marked by the creation of friendships that often lasts a lifetime, forming a key foundation of social support. It is also a time when young people tend to loosen ties with their parents in favor of emerging peer relationships (Sikora, 2012; Stanton-Salazar and Urso-Spina, 2005).

### 1.3 Global challenges and study aim

In recent years, the world has faced specific and unprecedented challenges related to the COVID-19 pandemic, compounded by the outbreak of the Russian–Ukrainian war. These two global events, largely unforeseen by most individuals, have become sources of immense stress (Kobylarczyk-Kaczmarek, 2023; Sikorska et al., 2021; Szredzińska and Włodarczyk, 2021; Cipora and Mielnik, 2022; Machniak, 2022). Such stress could be particularly pronounced among adolescents. Sudden changes in daily functioning, school closures, remote learning, and restrictions on leaving home were all factors that likely had a profound impact on teenagers. For this group, their understanding of the world is still developing and psychological defense mechanisms are often immature (Szredzińska and Włodarczyk, 2021; Pyżalski, 2021; Jaskulska et al., 2021). According to research, during this period, pre-existing mental health problems in adolescents tended to worsen, and many experienced mental health difficulties for the first time (Kobylarczyk-Kaczmarek, 2023; Sikorska et al., 2021; Szredzińska and Włodarczyk, 2021; Cipora and Mielnik, 2022; Machniak, 2022; Pyżalski, 2021). A significant increase in mental disorders was observed among school-aged youth. It is a trend that continues to rise (Kobylarczyk-Kaczmarek, 2023; Sikorska et al., 2021; Szredzińska and Włodarczyk, 2021; Poleszak and Katab, 2022). In such a challenging context, social support may have played a crucial protective role in reducing the risk of anxiety and depressive disorders (Kobylarczyk-Kaczmarek, 2023; Szredzińska and Włodarczyk, 2021; Pyżalski, 2021; Jaskulska et al., 2021; Poleszak and Katab, 2022; Maraszkiewicz, 2022). The aim of this study was to assess perceived social support and the severity of symptoms of depression and anxiety among students from Polish secondary schools in in the crisis situations of the COVID-19 pandemic and the war in Ukraine.

## 2 Procedure and participants

The study was conducted among secondary school students across the province of Silesian Voivodeship in Poland. Prior to data collection, written invitations to participate in the study were sent to 143 randomly selected secondary schools. School principals were informed in advance about the purpose and objectives of the study. From 143 schools, 56 agreed to take part in the project facilitated the distribution of the online survey to their students. The survey was administered electronically via a Google Form, which was sent to students through school email systems. Data were collected during a single data collection period from February 2022 to May 2022. The Google Form was constructed to accept only fully completed questionnaires. No distinction was made between specific sociopolitical or public health contexts (e.g., the COVID-19 pandemic or the war in Ukraine). However, it was noted in the introductory information that the study aimed to assess the relationship between the current well-being of the respondents and the context of difficult events such as the pandemic and the outbreak of the Russian-Ukrainian war.

A total of 1,456 students aged 14 to 19 participated in the study (*M* = 16.77, SD = 1.33), including 776 girls (*M* = 16.78, SD = 1.34) and 680 boys (*M* = 16.77, SD = 1.36). All respondents provided informed consent, and for those under the age of 18, parental consent was also obtained in accordance with ethical guidelines. Demographic data regarding the study group, divided into girls and boys, are presented in Table 1.

**Table 1:** Socio-demographic characteristics of the group (*n* = 1,456).

	Girls (*n* = 776)	Boys (*n* = 680)
	f (rf)	f (rf)
Place of residence		
Urban	429 (55.28%)	364 (53.53%)
Rural	347 (44.72%)	316 (46.47%)
Marital status		
Single	530 (68.30%)	517 (76.03%)
In a relationship	246 (31.70%)	161 (23.97%)
Smoking cigarettes/other form of nicotine		
Yes	149 (19.20%)	141 (20.74%)
Drinking alcohol		
Yes	285 (36.72%)	280 (41.18%)
Chronic diseases		
Yes	146 (18.81%)	98 (14.41%)
Psychiatric treatment history before pandemic		
Yes	90 (11.59%)	61 (8.97%)
Starting psychiatric treatment during the pandemic		
Yes	110 (14.18%)	49 (7.21%)
Form of preferential study		
Stationary	454 (58.51%)	373 (54.85%)
Online	322 (41.49%)	307 (45.15%)
The most important type of support in dealing with stress		
Support from mother	84 (10.8%)	45 (4.1%)
Support from father	9 (1.2%)	9 (1.3%)
Support from both parents equally	89 (11.5%)	98 (14.4%)
Support from siblings	22 (2.8%)	17 (2.5%)
Support from partners	150 (19.4%)	96 (14.1%)
Support from friends	206 (26.5%)	178 (26.2%)
Other form of support	216 (27.8%)	254 (37.4%)

F, frequency (number of cases); F(rf), relative frequency (%).

## 3 Measures

### 3.1 The multidimensional scale of perceived social support (MSPSS)

The multidimensional scale of perceived social support (MSPSS) takes into account the multidimensionality of perceived social support, considering three basic sources of support: a significant other, family and friends. The scale consists of 12 statements to which the respondent refers using a seven-point Likert scale, where 1 means “I strongly disagree” and 7 means “I strongly agree.” The scale was adapted to Polish conditions, and adaptation studies showed its satisfactory psychometric properties (Buszman and Przybyła-Basista, 2017).

### 3.2 Children’s depression inventory 2nd edition (CDI 2)

The instrument allows for the identification of children and adolescents suffering from depressive symptoms, and can also help in the diagnosis of clinical depression. As part of the Polish adaptation, standards were developed for people aged 7 to 18. The set of questionnaires includes versions intended for children and adolescents (self-assessment sheet), parents and teachers. The full version of the self-assessment sheet includes 28 items regarding depressive symptoms specific to a given developmental stage. The tool consists of the following subscales: emotional problems; negative mood; low self-esteem; problems in functioning; lack of effectiveness and interpersonal problems. Raw scores are converted into two standardized formats: a ten scale (ranging from 21 to 79) and a sten scale (ranging from 1 to 10). Both take into account the respondent’s age and gender, and in each case higher scores indicate greater severity of depressive symptoms. According to the ten scale, the following categories were distinguished: 21–39 (low), 40–59 (average), 60–64 (increased), 65–69 (high), and 70–79 (very high). In turn, the sten scale categories applied in this study were: 1–3 (low severity), 4–6 (moderate severity), and 7–10 (high severity) (Kovacs, 2015; Wrocławska-Warchala and Wujcik, 2017).

### 3.3 State–trait anxiety inventory (STAI)

The inventory contains 40 statements, half of which assess anxiety as a relatively stable personality trait (X2), and the remaining anxiety as a situationally conditioned state (X1). The instrument yields raw scores ranging from 20 to 80 for each subscale. These raw scores can be converted to standardized sten scores based on normative data to facilitate interpretation of anxiety levels (low, moderate, high). The sten scale ranges from 1 to 10. Sten 1–3 is defined as low; 4–7 as medium, and 8–10 as high. The higher the sten, the greater the severity of anxiety symptoms. The study used only a subscale assessing anxiety as a state (X1) The STAI (Spielberger, 1983) is a widely used self-report instrument designed to measure both state and trait anxiety in individuals aged 14 and older (Spielberger, 1983).

The author’s demographic data questionnaire included questions about gender, age, place of residence, type of school, preferred type of education, and psychiatric treatment. This questionnaire also includes definitions of the “significant other” which appears in the questionnaire MSPSS The term “significant other “was defined for participants as someone they would turn to first in times of distress and from whom they would expect support.

## 4 Statistical analysis

Standard statistical procedures were used in the analyses. The Kolmogorov–Smirnov test was used to assess the normality of distributions. Due to the fact that the distributions of variables deviated from the normal distribution, it was decided to use non-parametric methods in subsequent analyses. The Mann–Whitney U test was used to assess the significance of differences between the group of girls and boys in terms of variables such as perceived social support, and symptoms of depression and anxiety. The relationships between perceived social support and symptoms of depression and anxiety were analyzed separately for girls and boys. The Spearman rank correlation was used to examine the associations between the MSPSS total score and the CDI-2 and between the MSPSS total score and the STAI-X1, separately for girls and boys. The significance level of *α* < 0.05 was assumed as statistically significant. Calculations were performed in Statistical version 13.3.

## 5 Results

### 5.1 Description of the study group

In addition to socio-demographic data, the survey included a question about being in a relationship and the most important type of support. The following replies were received:

A. Both the majority of girls (68.30% (530)) and boys (76.03% (517)) were not in a permanent intimate relationship (Table 1).

B. Among girls, the largest number of respondents indicated support other than those mentioned (27.8%), support from friends (26.5%) and support from a partner (19.4%) as the most important in coping with stress. Among boys, the largest number of respondents indicated support other than those mentioned (37.4%), followed by the support of friends (26.2%) as the most important in coping with stress (Table 1). The respondents were also asked two separate questions about the stress they felt due to the COVID-19 pandemic and the stress related to the outbreak of the war in Ukraine. Respondents could answer on a scale of 0–10 points; where 0 – no sense of stress, and 10 – the greatest stress imaginable. In the boys’ population, the average COVID-19 stress score was 2.56 ± 2.58 SD points; while the average stress level result related to the outbreak of the war in Ukraine was assessed by the respondents as: 3.50 ± 2.89 SD points. The surveyed girls similarly scored: 4.31 ± 2.82 SD points and 5.46 ± 2.85 SD points. The cross-gender comparison using the Mann Whitney U test showed a statistically significant difference in stress related to the COVID-19 pandemic (Z = −12.227; *p* < 0.001), as well as stress related to the outbreak of the war in Ukraine (Z = −12.331; *p* < 0.001). In the case of stress related to the COVID-19 pandemic, girls (median = 4,000; Q1-Q3 = 2–6) showed higher stress levels compared to boys (median = 2,000; Q1-Q3 = 0–4). A similar trend was observed when comparing stress between girls (median = 6,000; Q1-Q3 = 3–8) and boys (median = 3,000; Q1-Q3 = 1–6) related to the outbreak of war in Ukraine. Before the pandemic, 8.97% (61) of surveyed boys and 11.59% (90) of girls were undergoing psychiatric treatment. During the COVID-19 pandemic, 7.21% (49) of surveyed boys and 14.18% (110) girls started psychiatric treatment (Table 1). A chi-square test was performed to assess the relationship between gender and the use of psychiatric treatment. In the case of initiation of psychiatric treatment before the indicated crisis event, no significant association with gender was noted (Chi-square = 2,691; *p* = 0.101). When psychiatric treatment was initiated after the onset of the COVID-19 pandemic, this tendency was observed to be significantly more common in girls (Chi-square = 18,096; *p* = 0.000).

### 5.2 Multidimensional scale of perceived social support (MSPSS)

Analyzing the results obtained in the MSPSS study group, the average overall score was 4.75 ± 1.51 points, in the family support subscale the average score was 4.38 ± 1.84 points, support from friends 4.90 ± 1.84 points, support from a significant other 4.98 ± 1.53 points. The differences between the group of girls and the group of boys were statistically significant in all the studied variables representing social support (Table 2).

**Table 2:** Differences between girls and boys in examined parameters.

	Girls (*n* = 776)			Boys (*n* = 860)			Mann–Whitney U	
	Median	Q1	Q2	Median	Q1	Q2	Z	*p*
MSPSS	5.167	4.000	5.958	4.917	3.750	5.833	−2.781	0.005
Support family	4.500	2.750	5.875	4.750	3.000	6.000	2.295	0.021
Support friends	5.750	4.000	6.500	5.000	3.250	6.250	−4.147	0.000
Support significant other	5.500	4.250	6.500	5.000	3.250	6.000	−6.789	0.000
CDI-2	19.000	12.000	26.000	14.000	8.000	21.000	−8.843	0.000
STAI X1	48.000	41.000	57.000	43.000	34.000	51.000	−9.262	0.000

MSPSS, Multidimensional Scale of Perceived Social Support; CDI-2, Children’s Depression Inventory 2; STAI-X1, The State–Trait Anxiety Inventory; Q1, lower quartile; Q3, upper quartile.

### 5.3 CDI-2 depressive symptoms rating scale

In the assessment of depression, the respondents scored an average of 17.36 ± 9.37 points. After dividing into groups according to gender, 19.44 ± 9.44 points were obtained, respectively for girls (61, increased depressive symptoms) and 14.99 ± 8.70 points for boys (56, average intensity of depressive symptoms). The difference between girls and boys groups in terms of the severity of depressive symptoms was statistically significant (Table 2).

### 5.4 Anxiety inventory – STAI-X1

In the anxiety assessment, the respondents scored an average of 46.39 ± 11.60 points. After dividing into groups according to gender, 49.08 ± 11.01 points were obtained, respectively for girls (sten 8 – high severity) and 43.33 ± 11.51 points for boys (sten 7, medium intensity). The difference between girls and boys groups in terms of the severity of anxiety as a state was statistically significant (Table 2).

### 5.5 Analysis of relationships between MSPSS, CDI-2, STAI-1

The Spearman rank correlations were used to examine the associations between the MSPSS total score and the CDI-2 and between the MSPSS total score and the STAI-X1, separately for girls and boys. Statistically significant negative correlations were observed between depressive symptoms (CDI-2) and anxiety symptoms (STAI-X1) and perceived social support (MSPSS), including its total score and all subscales, in both groups (see Tables 3, 4).

**Table 3:** Correlations between examined parameters – girls.

*n* = 776	MSPSS-global	Support from family	Support from friends	Support from significant other
CDI-2	−0.428*	−0.482*	−0.267*	−0.203*
STAI-X1	−0.331*	−0.360*	−0.232*	−0.166*

MSPSS, Multidimensional Scale of Perceived Social Support; CDI-2, Children’s Depression Inventory 2; STAI-X1, The State–Trait Anxiety Inventory; *Statistically significant with *p* < 0.05.

**Table 4:** Correlations between examined parameters – boys.

*n* = 680	MSPSS-global	Support from family	Support from friends	Support from significant other
CDI-2	−0.489*	−0.429*	−0.367*	−0.228*
STAI-X1	−0.417*	−0.502*	−0.339*	−0.289*

MSPSS, Multidimensional Scale of Perceived Social Support; CDI-2, Children’s Depression Inventory 2; STAI-X1, The State–Trait Anxiety Inventory; *Statistically significant with *p* < 0.05.

## 6 Discussion

### 6.1 Mental health during crises (COVID-19 and the war in Ukraine)

The changes that occurred in the surrounding reality during the COVID-19 pandemic were a source of significant stress for many people. Just when the world seemed to be returning to normal Russia’s aggression against Ukraine once again shocked the international community. During these two exceptional periods, a two-fold increase in the number of teenagers struggling with mental health problems was observed (Goto et al., 2024; Danese et al., 2025; Dąbkowska, 2020; Yoon and Kim, 2018). In our study, both girls and boys achieved high scores on the anxiety scale and average scores on the depression scale. Girls, however, scored higher on both scales than boys. Yoon et al. observed that affective symptoms tended to be more severe in women, which is consistent with epidemiological data indicating that women are more frequently diagnosed with affective and anxiety disorders (Chu et al., 2010). Men, on the other hand, are more frequently diagnosed with substance use disorders and schizoid personality disorder. Moreover, in our analysis, a higher percentage of girls than boys reported chronic illnesses and psychiatric treatment both before and during the pandemic (results were not statistically significant). Available research indicates that pre-existing psychiatric conditions and interventions may increase vulnerability to psychological stress during crises (Johansen et al., 2021; Pettit et al., 2011). However, it seems that it is not the fact of psychiatric treatment itself, but the previous mental health problems that led to it, that may make this group of teenagers more sensitive and prone to stress. In our study, adolescents of both sexes perceived the outbreak of war in Ukraine as a more stressful event than the ongoing COVID-19 pandemic. It is likely that the war represented a new and acute stressor, potentially triggering stronger emotional reactions than the prolonged, gradually easing restrictions related to the pandemic. The overall perceived stress level for both events was assessed by adolescents as moderate to low, with mean scores below the midpoint of the scale. It appears that the moderate level of stress associated with COVID-19 may have been caused by a significant easing of restrictions related to the pandemic, which has been ongoing for over 2 years. However, the outbreak of war may have seemed like an “abstract phenomenon” to young people, and its effects related to the influx of refugees from Ukraine during this period (February 2022–May 2022) were not yet felt.

### 6.2 Gender differences in anxiety, depression, and sources of support

The study showed that social support during crisis situations is more important for girls than for boys. Gender also differentiated the sources of support. The obtained results (statistically significant) showed that girls more often indicated that support from friends and significant other was important to them. “The significant other” was defined by participants as someone they would turn to first in times of distress and from whom they would expect support. Boys more often indicated family support as the most important. In the sociodemographic questionnaire, girls, on the other hand, indicated that mother’s support was particularly important to them, while boys indicated “other forms of support.” However, these results were not statistically analyzed. This may reflect gender-specific help-seeking behaviors and differences in emotional communication. The literature suggests that emotional support from peers and family has a protective effect on depressive symptoms in teenage girls (Dahlem et al., 1991; van der Wal et al., 2021). Boys, on the other hand, more often seek support in various types of activities or choose more instrumental support (Rueger et al., 2016).

### 6.3 Contextual factors: place of residence and protective role of social support

In our study, a significant percentage of participants lived in rural areas (approximately 45% of girls and 46% of boys). Rural youth may experience advantages associated with their place of residence, such as a close-knit community. Limited access to specialized mental health services may be a disadvantage. In such circumstances, informal support networks may become a particularly important coping resource when professional help is less accessible or less acceptable. The multifaceted relationship between place of residence and mental health was also discussed by Scardera et al. (2020). In our study, we also observed a relationship between perceived social support and scores on the CDI-2 and STAI-1 scales. The negative correlation between the aforementioned data may indicate a protective role of social support against anxiety and depressive symptoms. Similar results were obtained in the meta-analysis conducted by Jones et al. (2017) and the study by Cobham et al. (2020).

## 7 Conclusion

Social support during crisis situations was more important for girls than for boys in the group of Polish teenagers. Gender may have determined the sources of support.

Perceived social support may play an important role in coping with symptoms of anxiety and depression in crisis situations.

### 7.1 Study limitations

The presented study, like any study in which results were collected using the Internet, has certain limitations.

1. First, collecting data via an online form makes it impossible to monitor how the questionnaires are completed.
2. Using self-report questionnaires in the study is associated with the possibility of misunderstanding the instructions, and responses may be distorted by difficulties in the respondent’s self-assessment or unconscious manipulation.
3. Furthermore, the STAI questionnaire used in the study has not been validated in pediatric populations; the obtained results should be interpreted with caution.
4. The statistical methods used in the study only allow for the determination of the relationship between the parameters studied, not a cause-and-effect relationship.
5. Another limitation of this study is that the research material was collected exclusively in secondary schools, without creating control groups from other educational institutions at the same age.
6. Furthermore, the observed gender differences should be interpreted with caution, as they may be influenced by other unmeasured factors.
7. Another limitation of this study is the lack of data on the socioeconomic status of participants, which may influence mental health indicators.
8. Furthermore, the study did not differentiate between factors (the COVID-19 pandemic and the wars in Ukraine) that could have influenced participants’ emotional functioning.
9. The study is not longitudinal, so it is difficult to conclude whether the crisis situation had an impact on the mental state.
10. The term “significant other “was defined for participants as someone they would turn to first in times of distress and from whom they would expect support. The option “other form of support” did not allow for a precise assessment of the source of support.

## Data Availability

The original contributions presented in the study are included in the article/supplementary material, further inquiries can be directed to the corresponding author.
